# Sexually dimorphic oxytocin receptor-expressing neurons in the preoptic area of the mouse brain

**DOI:** 10.1371/journal.pone.0219784

**Published:** 2019-07-11

**Authors:** Kaustubh Sharma, Ryan LeBlanc, Masudul Haque, Katsuhiko Nishimori, Madigan M. Reid, Ryoichi Teruyama

**Affiliations:** 1 Department of Biological Sciences, Louisiana State University, Louisiana, United States of America; 2 Department of Molecular and Cell Biology, Graduate School of Agricultural Science, Tohoku University, Miyagi, Japan; University of Texas at El Paso, UNITED STATES

## Abstract

Oxytocin is involved in the regulation of social behaviors including parental behaviors in a variety of species. Oxytocin triggers social behaviors by binding to oxytocin receptors (OXTRs) in various parts of the brain. OXTRs are present in the preoptic area (POA) where hormone-sensitive sexually dimorphic nuclei exist. The present study was conducted to examine whether sex differences exist in the distribution of neurons expressing OXTRs in the POA. Using OXTR-Venus (an enhanced variant of yellow fluorescent protein) mice, the distribution of OXTR-Venus cells in the POA was compared between sexes. The total number of OXTR-Venus cells in the medial POA (MPOA) was significantly greater in females than in males. No detectable OXTR-Venus cells were observed in the anteroventral periventricular nucleus (AVPV) within the MPOA in most of the brain sections from males. We further examined the total number of OXTR-Venus cells in the AVPV and the rest of the MPOA between the sexes. The total number of OXTR-Venus cells in the AVPV in females (615 ± 43) was significantly greater than that in males (14 ± 2), whereas the total number of OXTR-Venus cells in the rest of the MPOA did not differ significantly between the sexes. Thus, the sexually dimorphic expression of OXTR-Venus specifically occurred in the AVPV, but not in the rest of the MPOA. We also examined whether the expression of OXTR in the AVPV is driven by the female gonadal hormone, estrogen. Immunocytochemistry and single-cell RT-PCR revealed the presence of the estrogen receptor α in OXTR-Venus cells in the female AVPV. Moreover, ovariectomy resulted in the absence of OXTR-Venus expression in the AVPV, whereas estrogen replacement therapy restored OXTR-Venus expression. These results demonstrate that the expression of OXTR in the AVPV is primarily female specific and estrogen dependent. The presence of the sexually dimorphic expression of OXTR in the AVPV suggests the involvement of OXTR neurons in the AVPV in the regulation of female-specific behavior and/or physiology.

## Introduction

The neurohypophysial hormone, oxytocin, is synthesized by magnocellular cells located primarily in the paraventricular (PVN) and supraoptic (SON) nuclei of the hypothalamus. The magnocellular cells send long axonal projections into the neurohypophysis where oxytocin is released into the general circulation in response to physiological demands, such as milk let down and parturition [1, 2]. The release of oxytocin also occurs within the brain and modulates many aspects of behaviors including but not limited to maternal care [3–9], female sexual behavior [10–12], male sexual behavior [10, 13, 14], pair/social bonding [15] [16, 17], aggression [18–20], anxiety [21], and fear [22, 23]. Oxytocin influences behaviors by binding to oxytocin receptors (OXTRs) that are widely distributed in various parts of the brain [24, 25].

The medial preoptic area (MPOA) is an essential component of the neural circuit that regulates maternal behavior [26–32]. Oxytocin acts on neurons in the MPOA to facilitate maternal behavior in rodents [28]. The action of oxytocin on the MPOA is also essential for the onset of maternal behavior at parturition in rats [6, 7]. The onset of maternal behavior is promoted by increased estrogen that facilitates the expression of OXTR in the MPOA [28]. These findings suggest that OXTR neurons in the MPOA are important neurons comprising a sexually dimorphic neural circuit that is associated with differences in parental care [33–37].

The present study was conducted to assess the sex differences in the distribution of OXTR neurons in the preoptic area (POA) using OXTR-Venus (an enhanced variant yellow fluorescent protein) mice. In contrast to transgenic reporter models, which use random integration of a reporter gene that could end up anywhere in the host genome, this OXTR-Venus mouse line is an OXTR knock-in model in which Venus is inserted into the locus exactly where OXTR is normally located [38]. Therefore, Venus likely achieves natural expression patterns and levels, while ectopic expression is less likely to occur. Unlike previously published reports on the localization of OXTRs in the brain that were conducted by either autoradiography of oxytocin binding [39–43] or *in situ* hybridization of OXTR mRNA [44], the use of OXTR-Venus mice provides a detailed distribution of OXTRs at the cellular level.

## Materials and methods

### Animals

OXTR-Venus mice in which a part of the OXTR gene was replaced with Venus (a variant of the yellow fluorescent protein) cDNA [38] were originally provided by Dr. Nishimori of the Tohoku University in Japan. A colony was established in a facility at Louisiana State University, and OXTR-Venus mice were backcrossed with C57BL6J mice for at least 10 generations. Four breeder pairs of OXTR-Venus heterozygous (+/-) male and female gave 22 litters of pups that were used for this study. Only virgin female and male mice (6–10 weeks old) were used. The males and females were housed in separate cages (maximum 4 mice/cage) in the same room on a 12:12 h light/dark cycle with access to food and water available *ad libitum*. For genotyping, genomic DNA was isolated from tail snips by incubating the tissues with REDExtract-N-Amp Tissue PCR Kit (Sigma, St. Louis, MO). The isolated DNA was subsequently genotyped using the following two sets of primers: F (5’-GTTGGGAACAGCGGTGATTA-3’) and R (5'-GGCTCAGGCTTTCTCTACTT-3'). All protocols and animal experiments were approved by the Institutional Animal Care and Use Committee at Louisiana State University.

### Immunocytochemistry

Mice (6–8 weeks old) were deeply anesthetized with Ketamine-Xylazine (9:1; 100 mg/kg; i.p.) and transcardially perfused with 0.01M sodium phosphate buffered saline (PBS; pH 7.2), followed by 4% paraformaldehyde in 0.1 M sodium phosphate buffer (PB; pH 7.2). The brains were extracted and postfixed in the same fixative for overnight. After infiltrated with 20% sucrose in 0.1 M PB for cryoprotection for 12 hr., coronal sections were transected at 40 μm by a sliding microtome (Leica SM2010R; Mannheim, Germany).

To enhance the signal of Venus, immunocytochemical localization of Venus with anti-green fluorescent protein (GFP) antibody (ab290; abcam, Cambridge, UK) was conducted in the brain sections obtained from adult virgin male and virgin female OXTR-Venus mice (8–10 week-old). The free-floating brain slices were incubated with the primary antibodies against GFP at dilutions of 1:10,000 in PBS containing 0.5% Triton X-100 (PBST) overnight with continuous gentle agitation at 4°C. The brain sections were subsequently incubated with a secondary antibody (goat anti-rabbit) conjugated with DyLight 594 (Jackson ImmunoResearch, West Grove, PA) for 4 hr at room temperature. The sections were mounted in polyvinyl alcohol (PVA) with anti-fading agent 1,4-Diazabicyclo[2.2.2]octane (DABCO) that consists of 4.8 g PVA, 12 g glycerol, 12 ml dH_2_O, 24 ml 0.2 M Tris-HCl and 1.25 g DABCO. The specificity of the anti-GFP antibody was tested on brain sections from wild type mice that do not express Venus. Immunoreactivity to Venus was not observed in the brain sections from wild type mice. Thus, the anti-GFP antibody specifically recognizes Venus, which is a variant of GFP. Fluorescence microscopy images (1280 x 1024) were acquired digitally (Eclipse 80i equipped with DS-QiMc, Nikon, Tokyo, Japan).

For double-fluorescence imaging of Venus and immunocytochemistry of estrogen receptor α (ERα), the brain sections were processed with polyclonal anti-ERα antibody (Cat#06–935; EMD Millipore, Billerica, MA) at dilutions of 1:8,000 in PBST overnight with continuous gentle agitation at 4°C. The sections were subsequently incubated with secondary antibody conjugated with fluorescence marker (AffiniPure Goat Anti-Rabbit IgG Alexa Fluor647; Jackson ImmunoResearch, West Grove, PA) at 1:400 dilution for 2–4 hr at room temperature. The sections were mounted in polyvinyl alcohol (PVA) with DABCO. Confocal fluorescence microscopy images (1,024 x 1,024; 1 μm optical section thickness) were acquired with a confocal microscope (Leica TCS SP2 spectral confocal microscope, Mannheim, Germany). Digital images were minimally altered in ImageJ software (Bethesda, NIH) with changes in dynamic range.

### Electrophysiology

#### Slice preparation

Female mice were deeply anaesthetized using Ketamine-Xylazine (9:1, 100 mg/kg i.p.) and perfused through the heart with cold artificial cerebral spinal fluid (ACSF) in which NaCl was replaced by equiosmolar sucrose. The brains were then removed and coronal slices (250 μm) containing the hypothalamic periventricular nucleus were collected using a vibrating microtome (Leica VT1200; Leica, Mannheim, Germany). The brain slices were kept in ACSF (in mM: 125 NaCl, 2.5 KCl, 1 MgSO_4_, 1.25 NaH_2_PO_4_, 26 NaHCO_3_, 20 D-glucose, 2 CaCl_2_, 0.4 Ascorbic acid, pH of 7.3–7.4, with an osmolality of 290–300 mOsm/kg H_2_O).

#### Whole cell patch clamp recording

OXTR-Venus neurons in the AVPV were identified with an epifluorescence microscope (Olympus BX50WI, Tokyo) equipped with a 40x water immersion lens (0.8 n.a.) and a CCD camera (ORCA-R^2^, Hamamatsu Photonics, Hamamatsu). Whole-cell membrane potential recordings were obtained with an Axopatch 700B amplifier (Molecular Devices, Foster City, CA). Traces were acquired digitally at 20 kHz and filtered at 5 kHz with a Digidata 1440A and in conjunction with PClamp 10 software (Molecular Devices, Foster City, CA). Patch electrodes were drawn with a Flaming/Brown micropipette puller (Model P-1000, Sutter Instrument Novato, CA) from borosilicate capillary glass tubing (1.1 mm ID, 1.5 mm OD, Sutter Instruments, Novato, CA) to have resistance of 4-8MΩ when filled with a pipette solution that contained (in mM): 140 K-Gluconate, 1 MgCl_2_, 10 HEPES, 10 CaCl_2_, 2 ATP(Mg^++^), and 0.4 GTP (Na^+^) and 11 EGTA. ACSF was saturated with 95% O_2_/5% CO_2_ and was warmed to 33–34°C during the recordings. Picrotoxin (100 μM) and 6,7-dinitro-quinoxaline-2,3(1H,4H)-dione (DNQX; 10 μM) were also added to ACSF to block the synaptic activity. The estimated liquid junction potential was +9.2mV; however, the data presented are not corrected for liquid junction potential.

#### Data analysis

To observe the effect of oxytocin on OXTR-Venus cells, membrane potentials were recorded in the presence or absence of oxytocin (100 nM) while no current was injected. The mean membrane potentials were obtained by averaging the membrane potential in 30 sec time frames. These time frames were captured at 30 sec before, 2 min after, and 3 min after the bath application of oxytocin. These sampling duration and points were selected because the peak response and plateauing of the membrane potential occurred at these time points in OXTR-Venus cells from heterozygous mice. The same sampling parameters were used to measure the membrane potential of OXTR-Venus cells from homozygous mice that did not show apparent changes in response to the application of oxytocin. An ANOVA with repeated measures was used to assess the changes in membrane potential in response to oxytocin. The difference in the amplitude of depolarization caused by application of oxytocin between heterozygous and homozygous mice was analyzed with the Student's *t* test. Differences were considered to be statistically significant at *p* <0.05. Box and whisker plots were used to represent numerical data: mean and median are represented by a filled circle and a line, respectively; the box extends to the quartiles of the data points; the whiskers extend to the furthest data points.

### Single-cell RT-PCR

#### Single cell harvest for single-cell RT-PCR

The brain slices were prepared as described above in Slice preparation / Electrophysiology. Tissues containing the AVPV were carefully dissected out using a razor blade, were incubated in oxygenated ACSF (35°C) containing Protease Type XIV (1.2 mg/ml: Sigma Chemicals, St Louis, MO, USA) for 20–30 min, and washed in a sodium isethionate solution consisting of (in mM): 140 sodium isethionate, 2 KCl, 4 MgCl_2_, 23 glucose, 15 HEPES, pH 7.3 (adjusted with 1M NaOH). The tissue was then triturated in sodium isethionate solution using three successively smaller fire-polished pasteur pipettes to dissociate OXTR-Venus cells. The supernatant containing dissociated neurons was transferred to a plastic Petri dish (Nunc, Rochester, NY, USA) and allowed to settle for approximately 5 min. Borosilicated glass capillary tubes (inner diameter 1.1 mm, outer diameter 1.5 mm Sutter Instruments, Novato, CA) were pulled on a Flaming/Brown micropipette puller (Model P-1000, Sutter Instrument Novato, CA) to make micropipettes (tip inner diameter of ~5 μm) for cell harvest. Dissociated OXTR-Venus cells were identified by an inverted microscope equipped with epifluorescence (Olympus IX5, Tokyo). Each identified OXTR-Venus neuron was individually harvested by a micropipette filled with RNase free water on a micromanipulator (MP225, Sutter Instrument, Novato, CA). After aspiration, the contents of the micropipette were ejected into an ice-cold 0.5 ml PCR tube and stored at -80°C.

#### RT-PCR

Harvested cells were subjected to one step RT-PCR using iTaq Universal SYBR green One-step Kit (Bio-Rad, Hercules, CA) according to manufacturer’s protocol with ABI ViiA-7 sequence detection system (ABI Applied Biosystem, Grand Island, NY). The sequences of primer sets used to detect OXTR was Forward 5'-GTGCAGATGTGGAGCGTCT-3' & Reverse 5'-AGAGATGGCCCGTGAAGAG-3' and ERα (ESR1) was Forward 5'-CTGCCAAGGAGACTCGCTAC-3' & Reverse 5'-GCAACTCTTCCTCCGGTTCT-3'. Identification of each cDNA of interest was based on the predicted size of each PCR product; 130 bp for OXTR and 179 bp for ERα.

### Ovariectomy and Estrogen replacement therapy

Siblings of the female mice (6–8 weeks old, weighing 20-23g) were randomly assigned to intact female, OVX, and OVX+E groups. These female mice were anesthetized with 1.5–2% isoflurane (4% for induction) in oxygen at a rate of 2 L/m, the ovaries were removed bilaterally (OVX), and received an osmotic mini-pump (ALZET model 1004, DURECT, Cupertino, CA) under the back skin two weeks after ovariectomy. Each mouse received a subcutaneous injection of buprenorphine hydrochloride (Buprenex, 0.1 mg/kg) after surgery to control pain. Each osmotic mini-pump contained either 13.9 mM 17β-estradiol (E2, E8875, Sigma-Aldrich, St. Louis, MO) dissolved in a solvent containing dimethyl sulfoxide (DMSO) and 70% Ethanol (1:4, respectively) or vehicle only. The concentration of E2 was estimated to release 10 μg of E2 per day at 0.11 μl/hr. After 2 weeks, animals were deeply anaesthetized using Ketamine-Xylazine (9:1, 100 mg/kg i.p.) and their brains were extracted and sectioned as described above in Immunocytochemistry. The groups of mice were euthanized on the same day to avoid age difference becoming a variable.

### Cell count and statistics

The group of OXTR-Venus neurons in the AVPV started appearing in the area surrounding the organum vasculosum laminae terminalis (OVLT) at the ventral end of the third ventricle (Bregma 0.5 mm in the mouse brain atlas) [45] and was continuously found in the next 22–25 consecutive 40 μm-thick coronal sections. The number of OXTR-Venus neurons in the AVPV was counted from the level of the OVLT (section -5) to the level of the suprachasmatic nucleus (SCN, section 25) where most of OXTR-Venus neurons were found. Average cell number per section is presented as the means ± SE. The Student's *t* test was used to compare the total number of OXTR-Venus neurons in the AVPV between sexes. The effects of OVX and E2 replacement therapy on the number of OXTR-Venus neurons were also analyzed by the Student's *t* test. Differences were considered to be statistically significant at *p* <0.05. Box and whisker plots were used to represent numerical data: mean and median are represented by a filled circle and a line, respectively; the box extends to the quartiles of the data points; the whiskers extend to the farthest data points.

## Results

### The validation of the anti-GFP antibody

**Fig 1:**
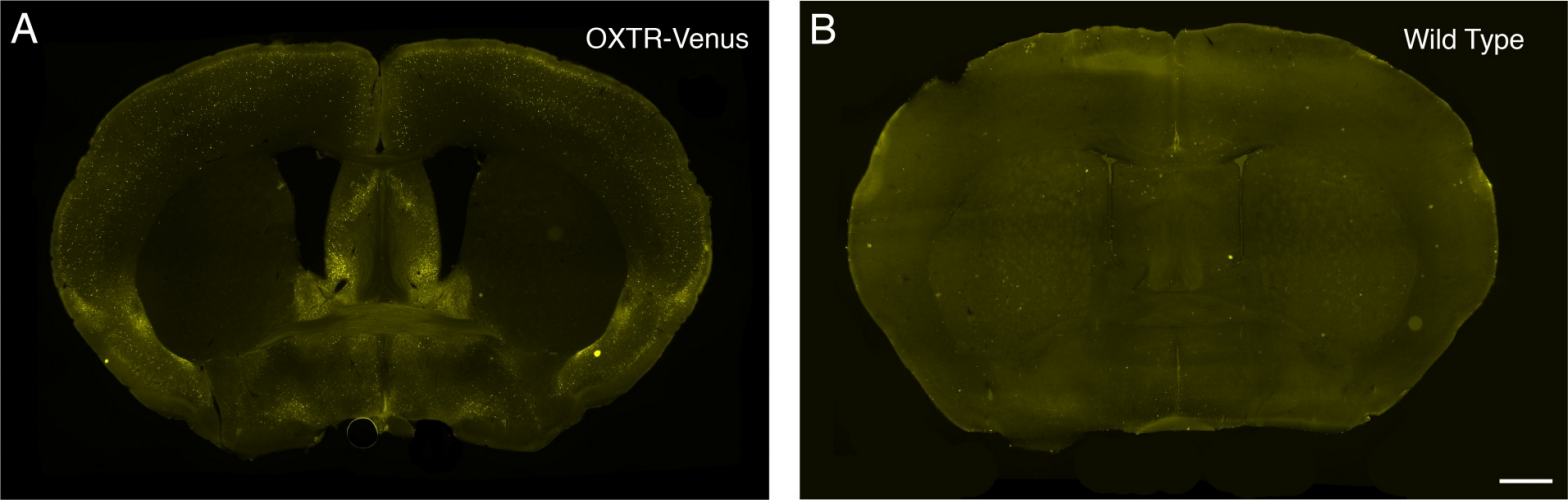
Fluorescent photomicrographs of coronal sections from a female OXTR-Venus mouse and a female wild type mouse. Immunocytochemistry using an anti-GFP antibody was conducted to enhance and preserve the fluorescent signal from Venus. **A.** Numerous immunoreactive OXTR-Venus cells were observed in various regions in the section from an OXTR-Venus mouse. **B.** No immunoreactive-cells were observed in the brain section from a wild type mouse. Scale bar = 1 mm.

To enhance and prolong the fluorescent signal of Venus (a variant of green fluorescent protein (GFP), immunocytochemistry was conducted using an antibody against GFP. Intense immunoreactive-cells were located in various part of the brain in sections from OXTR-Venus mice (Fig 1A); however, no immunoreactive-cells were observed in brain sections from wild type mice (Fig 1B). This finding suggests the antibody specifically recognized Venus in the brain sections.

### The overall distribution of OXTR-Venus cells in both sexes

Brains from young adult (6–8 weeks) virgin heterozygous (+/-) males (n = 6) and females (n = 8) were sectioned coronally from the beginning of the preoptic area to the end of the hypothalamus, and the distribution of OXTR-Venus cells was compared between the sexes. The overall distribution of OXTR-Venus cells was similar between sexes in all areas except within an area immediately along the third ventricle (3V) in the AVPV where OXTR-Venus cells were observed only in females, but not in males.

**Fig 2:**
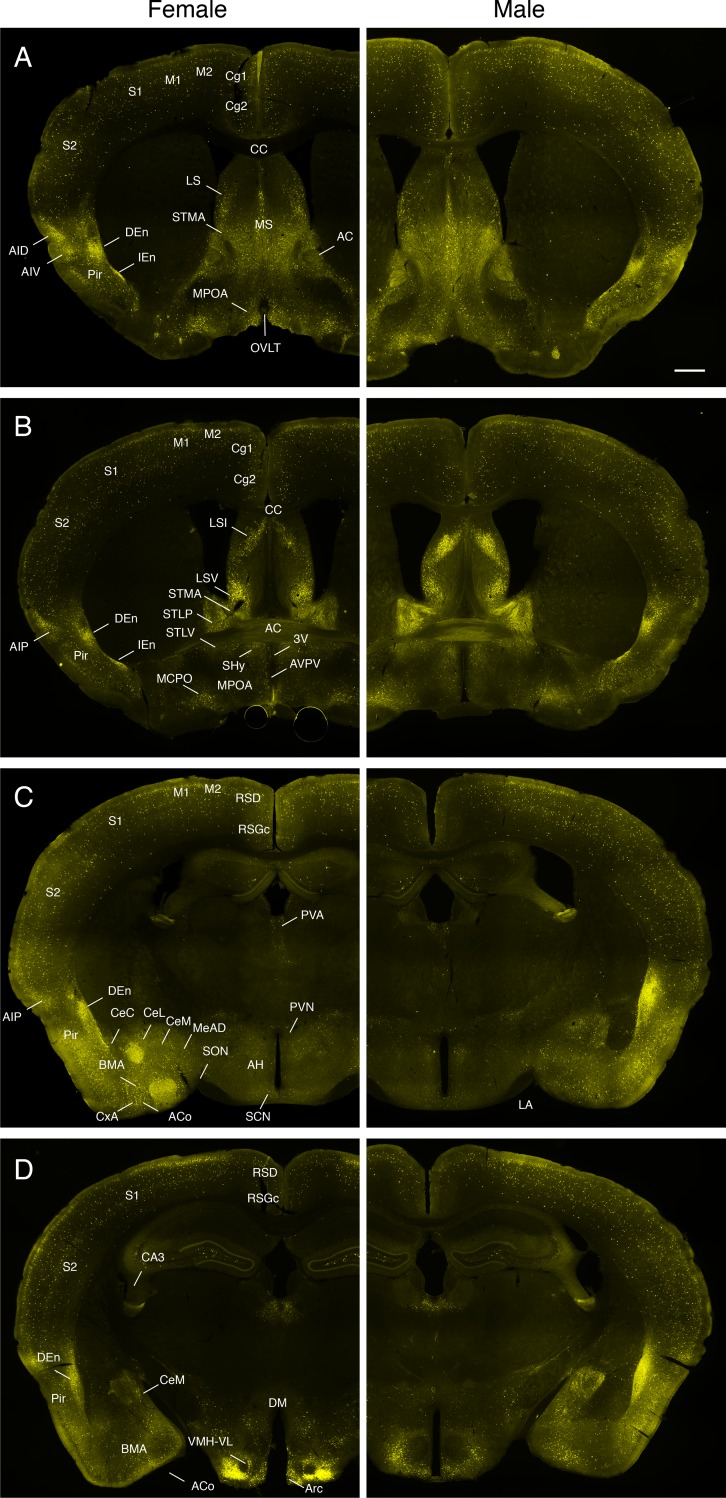
**Fluorescent photomicrographs showing OXTR-Venus immunoreactivity in coronal sections from a female (left panels) and a male (right panels) OXTR-Venus mouse. A**. At the level of OVLT, OXTR-Venus cells were found in the layers of the cortex (Cg1, Cg2, M1, M2, S1, and S2). Especially dense populations of OXTR-Venus cells were observed in the piriform cortex (Pir, DEn and IEn), insular cortex (AIP), lateral and medial septum (LS and MS), and anterior part of medial division of the bed nucleus of the stria terminalis (STMA). A moderate population of OXTR-Venus cells was found in the medial preoptic area (MPOA) surrounding the OVLT. **B**. At the level of the anterior commissure (AC), a dense population of OXTR-Venus cells was additionally found in the intermediate and ventral parts of the LS (LSI and LSV), and divisions of the BNST (STMA, STLP, and STLV). In the preoptic areas, magnocellular preoptic nucleus (MCPO) and Septohypothalamic nucleus (Shy) had a cluster of OXTR-Venus cells, whereas not so dense a population of OXTR-Venus cells was observed in the medial preoptic area (MPOA). A sexually dimorphic distribution of OXTR-Venus cells occurred in the AVPV where it appeared only in the female; however, the difference is not very clear due to low magnification of images. **C**. At the level of the supraoptic nucleus (SON), a prominent cluster of OXTR-Venus cells was additionally found in several areas in the amygdala (ACo, CxA, BMA, CeC, CeL, CeM, MeAD) and hypothalamus (AH, SCN, PVN). A sparse population of OXTR-Venus cells were also found in the thalamus (PVA). **D**. At the level of the posterior hypothalamus, a dense population of OXTR-Venus cells was observed in the VMH, while a sparse population was found in the CA1, CA2, and CA3 layers of the hippocampus. Overall, there is no obvious sex difference in the distribution of OXTR-Venus between sexes.

In coronal sections at the level of the organum vasculosum of the lamina terminalis (OVLT; Fig 2A), OXTR-Venus cells were sparsely distributed in the dorsal-lateral part of the cortex including the cingulate (Cg1 & 2), motor (M1 & M2), and somatosensory (S1 & S2) cortex. In contrast to the dorsal-lateral regions of the cortex, clusters of OXTR-Venus cells were found in several areas in the piriform (Pir) and insular cortex, namely the dorsal endopiriform claustrum (DEn), the intermediate endopiriform claustrum (IEn), the dorsal and ventral parts of the agranular insular cortex (AID & AIV). In the septum, lateral (LS) and medial (MS) regions along with the bed nucleus of the stria terminalis (BNST) medial division anterior part (STMA) had dense population of OXTR-Venus cells. A moderate population of OXTR-Venus cells were found in the MPOA surrounding the OVLT.

At the level of the anterior commissure (AC; Fig 2B), clusters of OXTR-Venus cells were found in the lateral septum (both intermediate (LSI) and ventral (LSV) parts) and the BNST (medial division anterior part (STMA), the posterior-lateral (STLP), and ventral-lateral (STLV) divisions). In the preoptic areas, the magnocellular preoptic nucleus (MCPO) and Septohypothalamic nucleus (Shy) had a cluster of OXTR-Venus cells, whereas a sparse population of OXTR-Venus cells was observed in the MPOA. A sexually dimorphic distribution of OXTR-Venus cells occurred in the AVPV where only females had a considerable number of OXTR-Venus cells.

At the level of the SON (Fig 2C), intense OXTR-Venus immunoreactivity was found in the amygdala along with areas in the insular and piriform cortex already described above. Within the amygdala, prominent OXTR-Venus cells were found in the anterior cortical amygdaloid nucleus (ACo), cortex amygdala transition (CxA), basomedial amygdala nucleus anterior (BMA), central amygdala (capsular region (CeC), lateral division (CeL), and medial division (CeM)), and medial amygdala n. anterior dorsal (MeAD). In hypothalamic regions, OXTR-Venus cells were sparsely distributed in the anterior hypothalamus (AH). In addition, a moderate population of OXTR-Venus cells was present in the suprachiasmatic nucleus (SCN). OXTR-Venus cells were not observed in the SON. In the PVN, OXTR-Venus cells were located in the dorsal cap region. In the thalamus, a group of OXTR-Venus cells was present in the paraventricular thalamic nucleus (PVA).

At the level of the posterior hypothalamus (Fig 2D), a large cluster of OXTR-Venus cells was found in the ventro-lateral division of the ventromedial hypothalamic nucleus (VMH-VL) and in the arcuate nucleus (Arc). A sparse population of OXTR-Venus cells was found in the dorsal medial hypothalamic nucleus (DM). In the hippocampus, OXTR-Venus cells were located in the CA3 field. The overall distribution of OXTR-Venus cells was largely comparable to that of OXTR detected in the C57BL/6J mouse brain by autoradiography of OXTR bindings [40, 46] indicating that the expression of Venus occurred in native cell types that express OXTR.

### The sexually dimorphic distribution of OXTR-Venus cells in the AVPV

**Fig 3:**
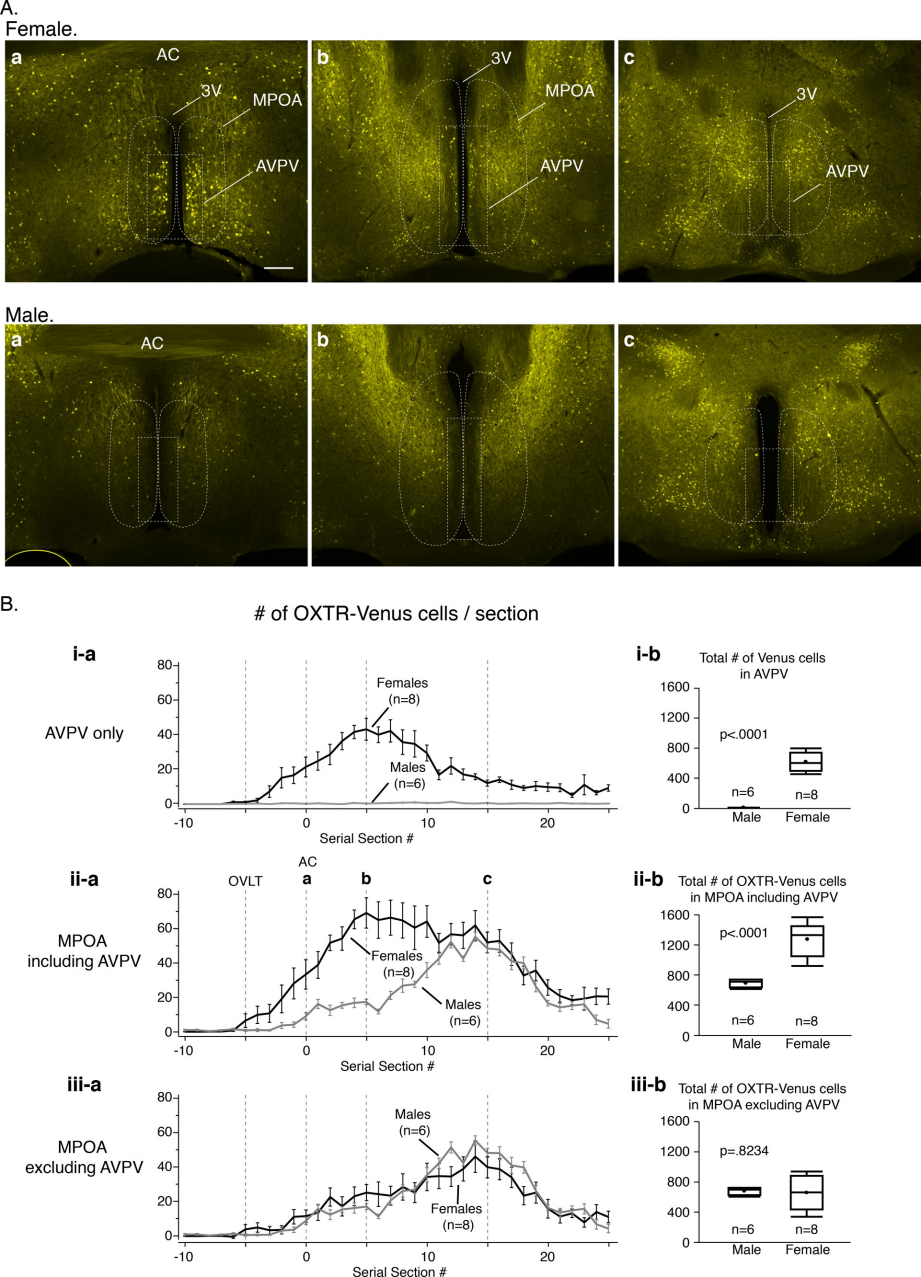
The sexually dimorphic distribution of OXTR-Venus cells in the AVPV. **A.** Fluorescent photomicrographs showing OXTR-Venus immunoreactivity in (**a**) the preoptic-hypothalamic regions at the level of the AC, (**b**) 5 sections (200 μm) posterior to the AC, and (**c**) 15 sections (600 μm) posterior to the AC from a female (top panels) and a male (bottom panels) OXTR-Venus mouse. **B**. The mean number of OXTR-Venus neurons in the AVPV (**i-a**), in the medial preoptic area (MPOA) including the AVPV (**ii-a**), and the MPOA without the AVPV (**iii-a**) on each brain section from 8 female and 6 male OXTR-Venus mice are plotted from the 10^th^ section anterior from the AC to the 25^th^ section posterior from the AC. The mean total number of OXTR-Venus cells in females was greater than in males in the AVPV (**i-b**) and in the MPOA including the AVPV (**ii-b**). There were no significant differences between sexes in the number of OXTR-Venus cells in the MPOA excluding the AVPV (**iii-b**). All numerical data are expressed as the mean ± SEM. AC: anterior commissure; POA: preoptic area. Scale bars = 200 μm.

The group of OXTR-Venus cells in the AVPV of females started to appear anteriorly at the level of the OVLT; however, the population became more prominent from the level of the anterior commissure (AC; Fig 3Aa) and was observed in the next 15 consecutive 40 μm sections posteriorly. The number of OXTR-Venus cells decreased considerably after 12–14 sections posteriorly from the AC.

To compare the distribution and number of OXTR-Venus cells between sexes, the number of OXTR-Venus cells in the AVPV in each brain section from 8 females and 6 males was counted and plotted (Fig 3Bi-a). The AVPV was defined as approximately the ventral two-third of an area within 130 μm from the edge of the 3V indicated by the dashed line in Fig 3A. The anterior-posterior coordinate of each brain section was adjusted by the presence of the AC; the first section containing the "connected" AC in the midline (Fig 3Aa) was designated as section #0 (Fig 3Bii-a), whereas the OVLT and the SCN were observed on #-5 and #25 sections, respectively. The mean number of OXTR-Venus cells in the AVPV on the anterior-posterior coordinated sections were plotted to better profile the population of the OXTR-Venus cells in females and males (Fig 3Bi). Significantly more OXTR-Venus cells in the AVPV were observed on sections #-3 to #25 of females than that of males (Fig 3i-a). Of six male mice examined, three mice had no detectable OXTR-Venus cell in their AVPV and the other three had 1 or 2 cells /section. The total number of OXTR-Venus cells in the AVPV was significantly greater in females (615 ± 43 cells) than in males (14 ± 2 cells) (Fig 3Bi-b; t_(12)_ = 11.76, p<0.0001).

To assess whether the sexually dimorphic distribution of OXTR-Venus cells extends into the area surrounding the AVPV, the number of OXTR-Venus cells in the MPOA was also counted. The mean number of OXTR-Venus cells/section was significantly higher in females than in males on sections #-3 to #10 (Fig 3ii-a). The total number of OXTR-Venus cells in the MPOA including the AVPV was significantly greater in females than in males (t_(12)_ = 6.16, p<0.0001); however, the number of OXTR-Venus cells was significantly higher in sections #0–10 where clusters of OXTR-Venus cells were observed in the AVPV (Fig 3Bii). The number of OXTR-Venus cells in the MPOA excluding the AVPV (Fig 3iii-a) was subsequently obtained by mathematical subtractions of Fig 3Bi-a from 3Bii-a. The number of OXTR-Venus cells was not significantly different on any sections between the sexes. Therefore, there was no sex difference in the number of OXTR-Venus cells in the MPOA excluding the AVPV (Fig 3Biii-b; t_(12)_ = 0.23, p = 0.823)

### The assessment of functional OXTR in OXTR-Venus cells in the AVPV

**Fig 4:**
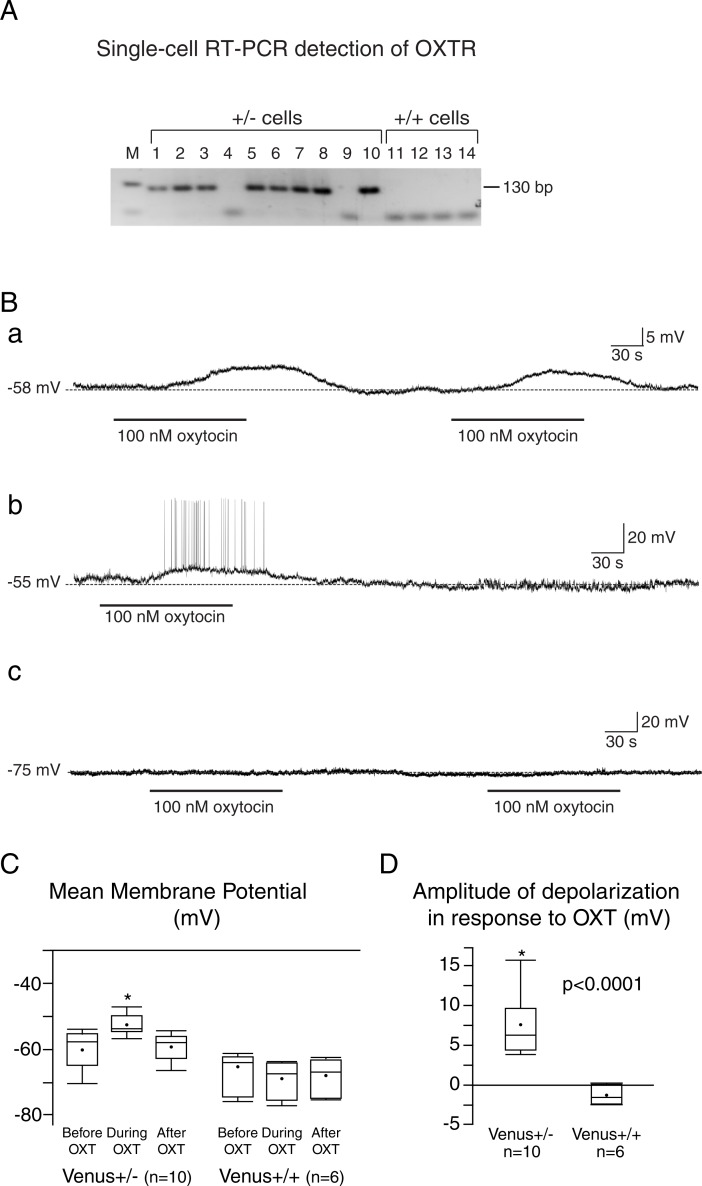
OXTR-Venus cells in the AVPV express functional OXTR. **A**. Single-cell RT-PCR analysis of OXTR expression in OXTR-Venus cells. Expression of OXTR mRNA was detected in 8 of 10 Venus cells harvested from OXTR-Venus heterozygous (+/-) females, but not detected from any of 4 OXTR-Venus cells obtained from homozygous (+/+) females. **B.** Examples of the effect of oxytocin on membrane potential and the firing pattern of OXTR-Venus (+/-) cells. **a**. Bath applications of oxytocin (100 nM) repeatedly caused membrane depolarization. **b**. The oxytocin-mediated depolarization caused repetitive firing. **c**. Application of oxytocin had no effect on OXTR-Venus (+/+) cells. **C.** In OXTR-Venus (+/-) cells, the mean membrane potentials before, during, and after the application of oxytocin (100 nM) were -60.36 ± 5.97 mV, -52.76 ± 3.47 mV, and -60 ± 3.74 mV, respectively. The application of oxytocin resulted in significant depolarization in OXTR-Venus (+/-) cells. In OXTR-Venus (+/+) cells, the mean membrane potentials before, during, and after the application of oxytocin (100 nM) were -67.57 ± 2.39 mV, -68.73 ± 2.35 mV, and -68.02 ± 2.24 mV, respectively. The application of oxytocin did not cause significant change in membrane potentials of OXTR-Venus (+/+) cells (F_(2,4)_ = 1.33, p = 0.19). **D.** The mean amplitude of depolarization in response to application of oxytocin was significantly (t_(14)_ = -5.35; p<0.0001) greater among OXTR-Venus (+/-) cells (7.6 ± 1.004 mV) than in OXTR-Venus (+/+) cells (-1.17 ± 1.3 mV).

Whenever a transgenic approach is used, it is always a concern that the expression of a transgene may interfere with the expression of a native gene or function of the native protein. In the OXTR-Venus mouse, a part of the *OXTR* was replaced by *Venus*. Therefore, the heterozygous (+/-) has a mono-allelic expression of both OXTR and Venus, while the homozygous (+/+) has bi-allelic expression of Venus without OXTR. Single-cell RT-PCR was performed to assess the expression of OXTR in OXTR-Venus neurons individually harvested from the dissected AVPV of heterozygous and homozygous OXTR-Venus female mice (Fig 4A). OXTR mRNA was detected from most of the heterozygous OXTR-Venus cells (38/48 cells from 8 virgin females), while OXTR mRNA was not detected from any homozygous OXTR-Venus cells (10 cells from 2 virgin females). In addition, to validate whether OXTR-Venus cells from heterozygotes produce functional OXTRs, we performed whole-cell patch-clamp recordings of OXTR-Venus cells in brain slices in ACSF containing DNQX and picrotoxin that blocked the synaptic activity. A bath application of oxytocin caused significant depolarization in all 10 OXTR-Venus cells examined in the AVPV from 5 female heterozygous OXTR-Venus mice (Fig 4Ba and 4C, F_(2,8)_ = 6.76, p = 0.0003). In some instances, the application of oxytocin induced depolarization that caused repetitive firing of action potentials (Fig 4Bb). A bath application of oxytocin did not, however, cause any measurable change in the membrane potential of all 6 cells examined from 5 female homozygous OXTR-Venus mice, which do not express OXTR (Fig 4Bc & 4C). The amplitudes of depolarization in response to application of oxytocin was significantly greater in heterozygous OXTR-Venus mice than in homozygous OXTR-Venus mice (Fig 4D).

### The assessment of estrogen dependency of OXTR expression in the AVPV

**Fig 5:**
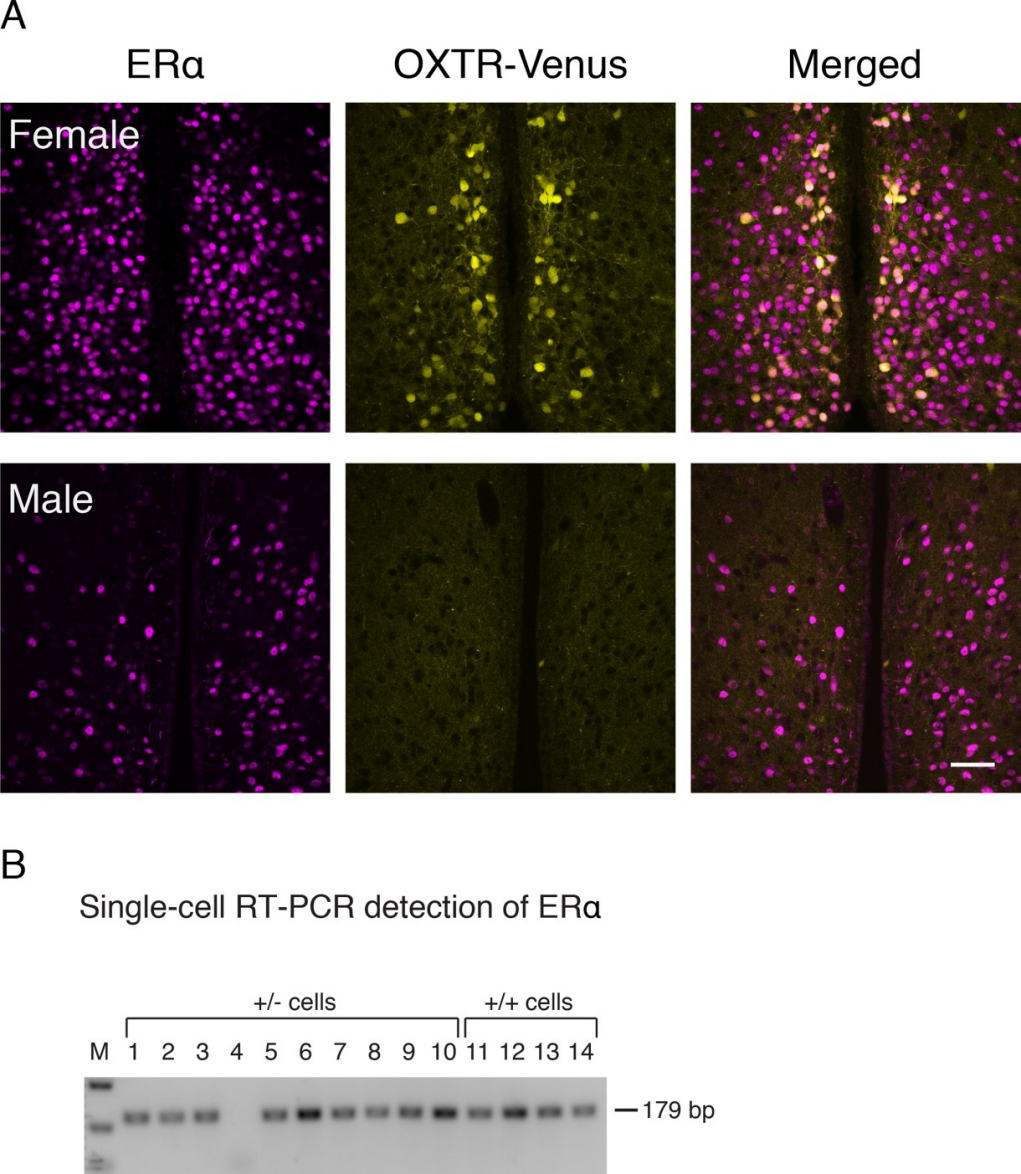
OXTR-Venus cells in the AVPV express ERα. **A.** Double confocal fluorescence photomicrographs of ERα immunoreactivity and OXTR-Venus from a female and a male OXTR-Venus (+/-) mouse. Many OXTR-Venus cells were seen in the AVPV from the female, whereas virtually no detectable OXTR-Venus cell was found in the AVPV from the male. The merged image shows that all OXTR-Venus cells were immunoreactive to ERα. **B**. Single-cell RT-PCR analysis of ERα expression in OXTR-Venus cells. The same set of cDNA derived from individually harvested OXTR-Venus cells used in Fig 2A was used. ERα mRNA was detected from all cells except cell #4.

Because the expression of OXTR-Venus cells in the AVPV occurred primarily in females, we hypothesized that expression of OXTR in the AVPV is supported by the female gonadal steroid, estrogen. To test this hypothesis, we conducted immunocytochemistry to examine whether the estrogen receptor α (ERα) is present in OXTR-Venus cells. ERα immunoreactive cells were found in the AVPV and its surrounding area of the MPOA in females and males (Fig 5A). Double fluorescence confocal microscopy revealed that all OXTR-Venus neurons were immunoreactive with ERα in females (Fig 5A). The presence of ERα in OXTR-Venus cells was also confirmed by single-cell RT-PCR (Fig 5B). Of 58 OXTR-Venus cells (48 cells from heterozygous and 10 cells from homozygous females) that were individually harvested from the AVPV, mRNA for ERα was detected in 57 cells. Moreover, ERα mRNA was detected in all the cells in which OXTR mRNA was detected. Thus, these results indicate that OXTR-expressing neurons in the AVPV also express ERα.

**Fig 6:**
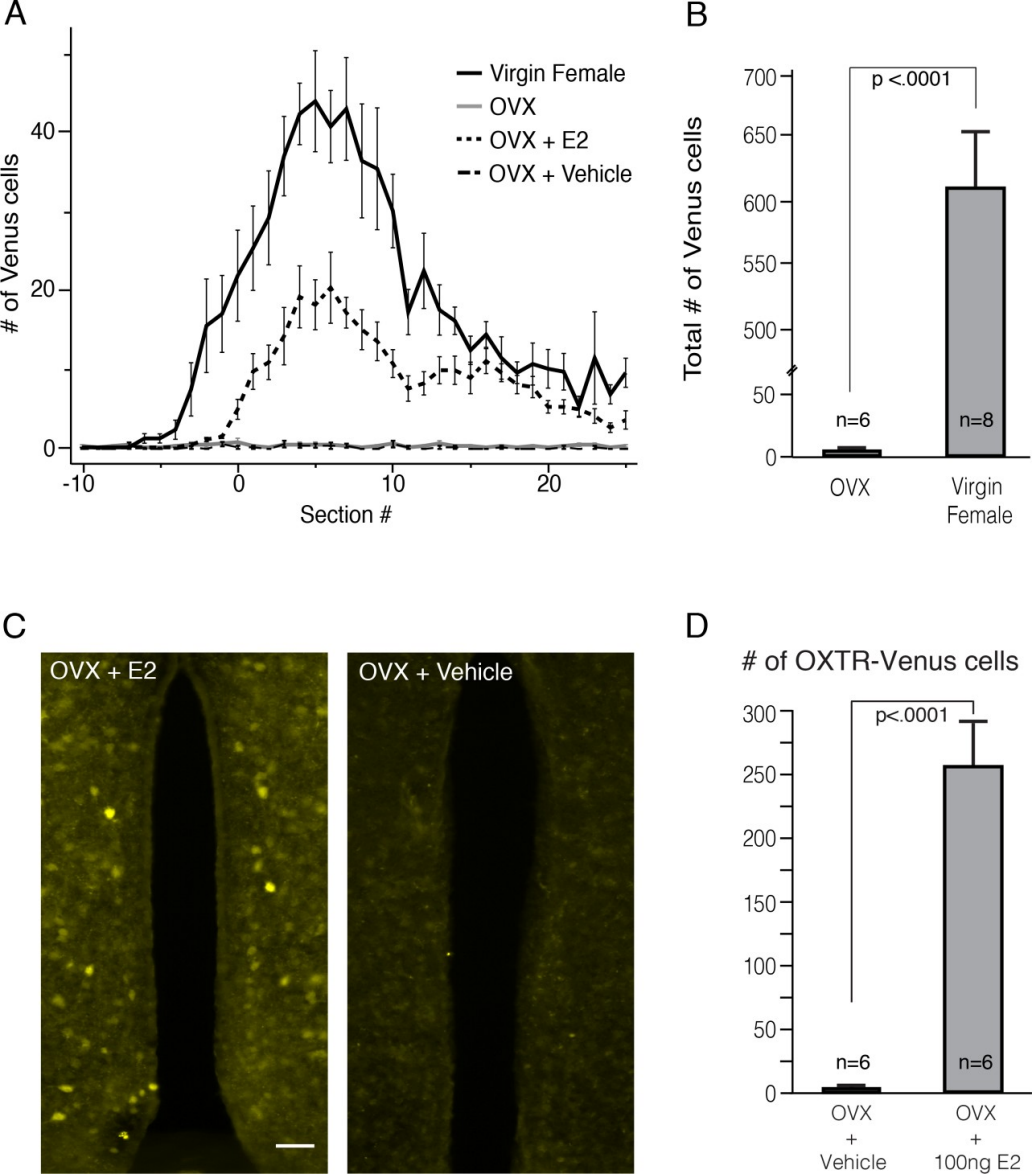
Expression of OXTR in the sexually dimorphic OXTR-neurons is supported by estrogen. **A.** Plot of the average number of OXTR-Venus neurons in the AVPV/section in the serial sections from the OVLT to SCN in intact, OVX, OVX+E2, and OVX+Vehicle female mice. **B.** The mean total number of OXTR-Venus cells in the AVPV from intact female and OVX. The mean total number of OXTR-Venus cells in OVX was significantly lower than that of females. **C.** Fluorescence microscopic images of OXTR-Venus cells in the AVPV from an OVX+E2 and an OVX+Vehicle. The group of OXTR-Venus cells observed in the AVPV in the intact females was not observed in OVX+Vehicle; however, the group of OXTR-Venus cells was observed in the OVX+ E2 mouse. **D.** The mean total number of OXTR-Venus cells in the AVPV of OVX+E2 was significantly higher than that of OVX+Vehicle. All numerical data are expressed as the mean ± SEM.

To test whether the expression of OXTR in the neurons of the AVPV depends on estrogen, we first ovariectomized (OVX) virgin female mice to remove the source of estrogen and examined whether the expression of OXTR-Venus was affected. The number of OXTR-Venus cells in the AVPV was significantly fewer in brain slices collected two weeks after ovariectomy (n = 6, 7.3 ± 2.25 cells) compared to that of intact virgin female siblings (n = 8, 614.9 ± 43.2 cells, Fig 6A & 6B; t_(12)_ = -12.04, p<0.0001). Next, we tested whether estradiol (E2) replacement therapy would restore the population of OXTR-Venus cells. Significantly more OXTR-Venus cells were observed in the AVPV of OVX mice that received two weeks of E2 therapy (OVX+E2; 100ng/day; n = 6, 147.6 ± 14.9 cells) compared to OVX mice that received vehicle only (OVX+ Vehicle; n = 6, 5.92 ± 1.24 cells; Fig 6C & 6D; t_(10)_ = 8.25, p<0.0001). These findings suggest that expression of OXTR in the cells of the AVPV is supported by estrogen.

## Discussion

### Sexually dimorphic distribution of OXTR in the AVPV

Most of the previously published reports on the localization of OXTRs in rodent brains were conducted by *in vitro* receptor autoradiography with a selective radiolabeled OXTR ligand [39, 40, 42, 43, 46–55] or autoradiography of *in situ* hybridization of OXTR mRNA [44, 56]. However, none of these studies reported the presence of OXTR binding in the AVPV. Autoradiography lacks the ability to identify the precise distribution of OXTRs at the cellular level. Thus, the lack of detection of OXTR binding in the AVPV may be due to the marginal size of the AVPV which may be difficult to identify autoradiographically.

OXTR binding studies demonstrated that the distribution of OXTR binding is both brain region-specific and species-specific (reviewed in [57]). Whereas the species difference in the distribution of OXTR binding suggests the species-specific regulation of behavior by oxytocin, the sex differences in OXTR binding were also found in region- and species-specific manner. For example, sex-differences in the distribution of OXTR binding were found in various forebrain regions and within the hypothalamic VMH in rats [39, 47]; however, such sex-differences were not observed in C57BL/6J mice [46]. The present study did not quantitatively analyze the density of OXTR-Venus cells in each brain area except the AVPV and MPOA, because such experiments were not within the scope of the study. Therefore, the sex-difference in the density of OXTR-Venus cells in other brain regions are yet to be determined. However, in stark contrast to all other brain areas where OXTR-Venus cells were found in both sexes, the presence of OXTR-Venus cells in the AVPV occurs nearly exclusively in females. Sex-differences in the distribution of OXTR cells in the AVPV are unique and strongly indicate the involvement of OXTR cells in the AVPV in the regulation of oxytocin induced sex-specific behaviors and/or physiology.

### Estrogen-dependent expression of OXTR in the AVPV

Estrogen increases OXTR gene transcription [58] in the uterus [59] and brain [60]. A study also showed that the treatment with estrogen resulted in significant increase in OXTR binding in the MPOA and lateral septum of virgin female rats [61]. The estrogen-induced expression of OXTR in the brain is, at least partly, mediated by the ERα [62]. Estrogen-ERα regulates transcription of the OXTR gene through CG-rich SP1 transcription factor binding sites [63]. These findings imply that DNA methylation affects the interaction between SP1, ERα and their binding sites in the OXTR promoter [64]. The present studies found all OXTR-Venus cells in the AVPV were ERα-immunoreactive. Moreover, the expression of OXTR in the AVPV is clearly estrogen dependent, because OXTR-Venus was not observed in the AVPV of ovariectomized females, whereas OXTR-Venus expression was restored in OVX that received estrogen therapy. Interestingly, OXTR expression in numerous other locations were not affected by OVX (data not shown) indicating not all OXTR cells in the brain are estrogen dependent.

### Possible role of OXTR cells in the AVPV

The AVPV is a small cell group located immediately surrounding the area of the ventral half of the third ventricle at the anterior extreme. The AVPV is surrounded by the MPOA. Both the AVPV and MPOA are known to be sexually dimorphic areas [65–68]. The onset of maternal behavior at parturition in rats requires activation of OXTR in the MPOA [6, 7] by central oxytocin release that is activated by vaginocervical and suckling stimulations [69–71]. The MPOA neural activity is also necessary for maintenance of maternal behavior [28]. Moreover, it is speculated that one of the functions of estrogen is to stimulate the expression of OXTRs so that the critical neurons become responsive to oxytocin thus allowing oxytocin to activate MPOA that regulates maternal behavior [28]. While these studies demonstrated the importance of OXTR in the general area of the MPOA for the regulation of maternal behavior, these previous investigations did not provide a detailed neural structure that expresses OXTR. Therefore, the presence of estrogen-dependent and sexually dimorphic distribution of OXTR neurons in the AVPV implies that OXTR-neurons in the AVPV are involved in the induction of maternal behavior; however, behavioral studies using specific knock-out of OXTR in the AVPV is required to confirm such a hypothesis.

Several female-biased sexually dimorphic characteristics in the AVPV were previously documented. A markedly larger number of tyrosine hydroxylase-immunoreactive (TH^+^: putative dopaminergic) neurons were found in the AVPV of females than in males [65, 72]. More recently, the ablation of TH^+^ neurons in the AVPV was found to impair maternal behavior whereas optogenetic stimulation or increased tyrosine hydroxylase expression in these cells was found to enhance maternal care [73]. Moreover, TH^+^ neurons in the AVPV relay a monosynaptic input to oxytocin neurons in the PVN [73], suggesting that stimulation of oxytocin neurons by TH^+^ neurons from the AVPV is involved in induction of maternal behavior in females. Thus, it is possible that OXTR neurons interact with TH^+^ neurons within the AVPV to generate a positive feedback loop between TH^+^ neurons and oxytocin neurons to drive maternal behavior during pregnancy and lactation when the circulating level of estrogen is naturally high [74]. Interestingly, the same treatments, optogenetic stimulation or over expression of tyrosine hydroxylase in TH^+^ neurons, had no effect on parental care in males [73]. Thus, the lack of parental behavior in male mice to the extent of females [75–77] may be due to the absence of OXTR neurons in the AVPV.

## Conclusions

The present study demonstrated the sexually dimorphic distribution of OXTR neurons in the AVPV in the mouse brain. The presence of OXTR-Venus neurons in the AVPV is nearly exclusive to females. The expression of OXTR in the AVPV is estrogen dependent, as ovariectomy resulted in the absence of OXTR-Venus, whereas estrogen replacement therapy restored the expression of OXTR-Venus. The estrogen dependent expression of OXTR may be mediated by ERα as ERα immunoreactivity was observed in all OXTR-Venus cells in the AVPV; however, this notion must be confirmed by specific manipulation of ERα activity in these OXTR neurons. The functional significance of sexually dimorphic OXTR neurons in the AVPV is currently unknown; however, because the onset of proper maternal behavior at parturition requires activation of OXTR in the MPOA, the female specific expression of OXTR in neurons of the AVPV implies that these neurons are involved in the induction of maternal behavior. Further studies are necessary to elucidate the role of the OXTR neurons in behavior and physiology.

## Supporting information

S1 Data OXTR-Venus cell counting raw data file. (XLSX)

S2 Data Electrophysiology raw data file. (XLSX)
